# Determining Surgical Complications in the Overweight (DISCOVER): a multicentre observational cohort study to evaluate the role of obesity as a risk factor for postoperative complications in general surgery

**DOI:** 10.1136/bmjopen-2015-008811

**Published:** 2015-07-20

**Authors:** Dmitri Nepogodiev, Stephen J Chapman, James Glasbey, Michael Kelly, Chetan Khatri, Thomas M Drake, Chia Yew Kong, Harriet Mitchell, Ewen M Harrison, J Edward Fitzgerald, Aneel Bhangu

**Affiliations:** 1 University of Birmingham, School of Cancer Sciences, Birmingham, UK; 2 St James University Hospital, Leeds, UK; 3 University Hospital of Wales, Cardiff, UK; 4 Aintree University Hospital, Liverpool, UK; 5 Imperial College London Medical School, London, UK; 6 University of Sheffield Medical School, Sheffield, UK; 7 University of Glasgow Medical School, Glasgow, UK; 8 University of Bristol Medical School, Bristol, UK; 9 Department of Surgery, University of Edinburgh, Edinburgh, UK; 10 University College London, London, UK; 11 University of Birmingham, School of Cancer Sciences, Birmingham, UK

**Keywords:** SURGERY

## Abstract

**Introduction:**

Obesity is increasingly prevalent among patients undergoing surgery. Conflicting evidence exists regarding the impact of obesity on postoperative complications. This multicentre study aims to determine whether obesity is associated with increased postoperative complications following general surgery.

**Methods and analysis:**

This prospective, multicentre cohort study will be performed utilising a collaborative methodology. Consecutive adults undergoing open or laparoscopic, elective or emergency, gastrointestinal, bariatric or hepatobiliary surgery will be included. Day case patients will be excluded. The primary end point will be the overall 30-day major complication rate (Clavien-Dindo grade III–V complications). Data will be collected to risk-adjust outcomes for potential confounding factors, such as preoperative cardiac risk. This study will be disseminated through structured medical student networks using established collaborative methodology. The study will be powered to detect a two-percentage point increase in the major postoperative complication rate in obese versus non-obese patients.

**Ethics and dissemination:**

Following appropriate assessment, an exemption from full ethics committee review has been received, and the study will be registered as a clinical audit or service evaluation at each participating hospital. Dissemination will take place through national and local research collaborative networks.

## Background

Obesity has reached ‘epidemic’ levels across the world, challenging healthcare systems and economies in developed and developing countries. Obesity rates in the UK have risen dramatically in the last decade, from 13.2% to 24.4% in males, and 16.4% to 25.1% in females.^1^ In UK surgical practice, 30% of patients are identified as obese.^2^

Obesity is a known risk factor for several medical morbidities, including cardiovascular disease and diabetes. It has also been associated with an increased risk of several malignancies, including cancer of the colon and oesophagus.^3^ Conflicting evidence exists regarding the impact of obesity on postoperative complications following gastrointestinal surgery. A study of over 6 000 patients demonstrated no difference in mortality and postoperative morbidity for obese and non-obese patients.^4^ Recent studies have identified an obesity paradox, with moderate obesity offering protection from adverse events, whereas underweight patients are at greater risk.^5^ ^6^ However, other reports have suggested obesity is associated with an increased risk of surgical-site infection and venous thromboembolism.^7–9^

### The need for further evidence

Most studies exploring the role of obesity in determining postoperative complication rates have either been single centre, retrospective cohort studies or secondary analyses of previously collected data. There is a need for a multicentre prospective study that is primarily designed to address whether obesity is associated with an increased risk of postoperative complications. Detailed patient background should be collected in order to risk-adjust outcomes for potential confounders such as preoperative cardiac risk and socioeconomic status.

### Primary aim

The primary aim of the Determining Surgical Complications in the Overweight (DISCOVER) study is to determine whether obesity is associated with an increased risk of postoperative complications following gastrointestinal, bariatric and hepatobiliary surgery.

### Hypothesis

The 30-day major postoperative complication rate, following risk adjustment, should be equivalent in obese and non-obese patients.

## Methods

### Study design

**Figure 1:**
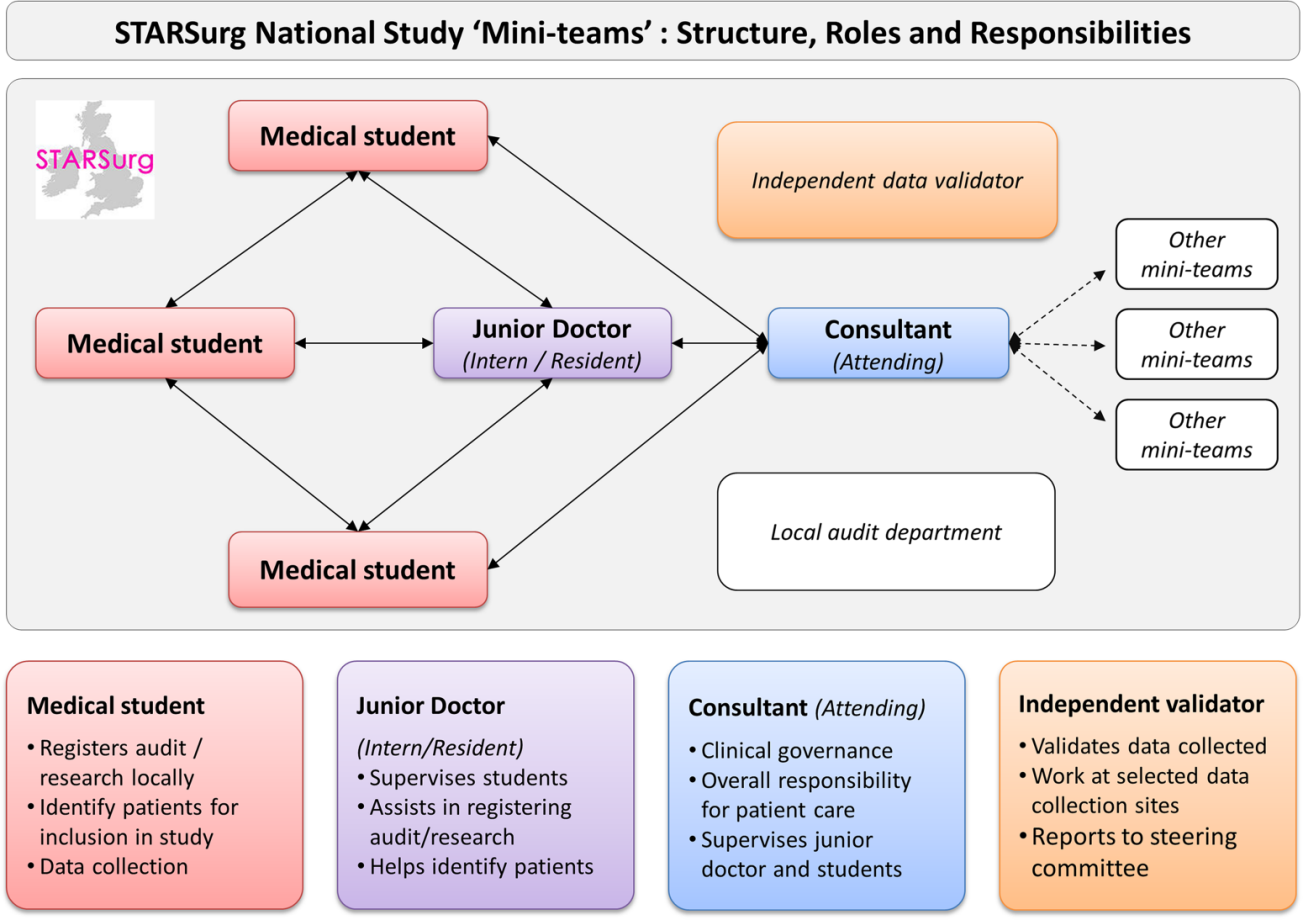
STARSurg ‘Mini-Team’ structure, roles and responsibilities.

A national multicentre prospective cohort study disseminated through collaborative university medical school and student networks (figure 1). The generic collaborative methodology has been described previously.^10^

### The STARSurg network

Student Audit and Research in Surgery (STARSurg) is the UK's national medical student research collaborative coordinated by a team of medical students and supervisors. Given common problems faced by students wishing to engage in high-quality extracurricular academic projects,^11^ the STARSurg network was formed to empower participation by forming links with supervising junior doctors and consultants. Through this, students contribute data to national studies while gaining an understanding of clinical academia, audit and research methodology and ethical considerations. This network facilitates multicentre, student-led audit and research projects with the ultimate aim of engaging students early in academic projects in order to help embed audit and research as a fundamental aspect of routine clinical practice. The educational model used and the benefits that participating students can derive from this have been described previously.^12^

The STARSurg network has previously delivered the world's first student-led multicentre study. Over 250 medical students and 100 junior doctors participated across 109 hospital centres to collect data on over 1500 patients undergoing gastrointestinal resection to investigate non-steroidal anti-inflammatory drugs (NSAIDs) as risk factors for postoperative adverse events.^13^ The specific methodology for this network has been previously described in the literature.^14^

### Study setting

This study will take place in general surgical units in state (publically funded) hospitals in the UK and the Republic of Ireland. Any hospital performing elective or emergency gastrointestinal, bariatric or hepatobiliary surgery may participate. Each centre will contribute up to three 2-week sets of consecutive patient data.

### Inclusion criteria

- Consecutive adult patients undergoing gastrointestinal, bariatric or hepatobilliary surgery.
- Patients undergoing either elective or emergency, and open, laparoscopic, laparoscopic-assisted or laparoscopic-converted procedures may be included.

### Exclusion criteria

- Patients under 18 years of age
- Day case surgery (ie, patients without an overnight hospital stay immediately preceding or following their operation)
- Hernia surgery, unless bowel resection is performed
- Minor anorectal procedures, unless there is an abdominal or laparoscopic approach
- Transplant surgery
- Trauma indication
- Gynaecological primary indication
- Urological primary indication
- Vascular primary indication

The primary end point of DISCOVER is major postoperative complications requiring reoperation, unplanned admission to intensive care or resulting in death. To ensure accurate case ascertainment, collaborators’ workloads have been rationalised by only including those procedures that commonly result in major complications. Consequently, low-risk day case, hernia and anorectal surgery have been excluded.

The audit standard in this study is that all patients should have their body mass index (BMI), calculated on admission to hospital, reflecting current guidelines from the National Institute for Health and Care Excellence (NICE).^15^

### Primary outcome measure

The primary outcome measure will be the 30-day major postoperative complication rate. Major complications will be defined as Clavien-Dindo grade III–V complications. The Clavien-Dindo classification has been selected as the primary outcome measure as it is a clinically relevant, an internationally standardised and validated scoring system for postoperative complications (table 1).^16^ It is based on the interventions required to treat complications, taking a holistic account of clinically significant events.

**Table 1:** The Clavien-Dindo classification of postoperative complications

Grade	Definition (examples listed in italics)
I	Any deviation from the normal postoperative course without the need for pharmacological (other than the ‘allowed therapeutic regimens’), surgical, endoscopic or radiological intervention Allowed therapeutic regimens are: selected drugs (antiemetics, antipyretics, analgesics, diuretics and electrolyte replacement), physiotherapy and wound infections opened at the bedside but **not** treated with antibiotics *Examples: Ileus (deviation from the norm); hypokalemia treated with sando K; nausea treated with cyclizine; acute kidney injury treated with intravenous fluids*
II	Requiring pharmacological treatment with drugs beyond those allowed for grade I complications. Blood transfusions and total parenteral nutrition are also included *Examples: Surgical site infection treated with antibiotics; myocardial infarction treated medically; Deep venous thrombosis treated with enoxaparin; pneumonia or urinary tract infection treated with antibiotics; blood transfusion for anaemia*
III	Requiring surgical, endoscopic or radiological intervention *Examples: Return to theatre for any reason; therapeutic endoscopic therapy (do not include diagnostic procedures); interventional radiology procedures*
IV	Life-threatening complications requiring critical care management; neurological complications including brain haemorrhage and ischaemic stroke (excluding TIA). *Examples: Single or multiorgan dysfunction requiring critical care management, e.g. pneumonia with ventilator support, renal failure with filtration; SAH; stroke*
V	Death of a patient

TIA, transient ischaemic attack.

### Secondary outcomes

Secondary outcome measures will be rates of system-specific complications (table 2), unplanned admission to the critical care unit, reoperation and readmission.

**Table 2:** System-specific complication outcome measures

Cardiovascular	
Angina (exacerbation)	Increase in chest pain requiring start or increase of medications
Arterial thrombosis/embolism	Include peripheral arterial thrombosis or embolism (not including stroke) (not including stroke) demonstrated by CT, MRI or angiography
Arrythmia	Any cardiac arrhythmia demonstrated on an ECG, except sinus tachycardia and sinus arrhythmia
Hypertension	Increase in systolic blood pressure requiring start or increase of medications
Myocardial ischaemia	Include ST-elevation myocardial infarction (STEMI), non-ST-elevation myocardial infarction (NSTEMI) and unstable angina. Diagnosis must have been confirmed following review of the patient by a cardiologist/on-call medical team
Venous thrombosis, deep vein thrombosis (DVT)	Peripheral venous thrombosis demonstrated by ultrasound, CT, MRI or angiography
Venous thrombosis, other	Venous thrombosis of the abdominal venous systems, including the coeliac, splenic, hepatic and mesenteric veins. Thrombosis should be demonstrated by CT or MRI
Metabolic	
Hypoglycaemia	Low blood sugar requiring intervention
Hyperglycaemia	High blood sugar requiring increase or start of new medications
Hypokalaemia	Low serum potassium requiring intervention
Hyperkalaemia	High serum potassium requiring intervention
Hypomagnesaemia	Low serum magnesium requiring intervention
Hyponatraemia	Low serum sodium requiring intervention. Include syndrome of inappropriate antidiuretic hormone secretion (SIADH)
Hypernatraemia	High serum sodium requiring intervention
Hypophosphatemia	Low serum phosphate requiring intervention
Neurological	
Head injury	Include extradural haemorrhage, subdural haemorrhage, subarachnoid haemorrhage, cerebral contusion demonstrated on CT or MRI
Stroke/TIA,	Include transient ischaemic attack (TIA), ischaemic or haemorrhagic stroke. Diagnosis must have been confirmed following review of the patient by a stroke physician/on call medical team
Renal	
Acute kidney injury	Acutely deranged renal function, with serum creatine increased to at least 1.5 times greater than the most recent preoperative baseline
Urinary retention	Failure to pass urine, requiring urinary catheterisation
Urinary tract infection (UTI)	The patient has had clinical evidence of urinary tract infection. UTI must be proven by mid-stream/catheter specimen culture
Respiratory	
Acute respiratory distress syndrome (ARDS)	Respiratory failure not explained by cardiac failure or fluid overload, with chest radiograph or CT scan demonstrating bilateral opacities not fully explained by effusions, lobar/lung collapse or nodules
Atelectasis	Collapse of part of the lung, confirmed by chest X-ray or CT scan
Haemothorax	Presence of blood in the pleural space, confirmed by chest X-ray or CT scan
Pleural effusion	Presence of fluid in the pleural space, confirmed by chest X-ray or CT scan
Pneumonia, aspiration	Pulmonary inflammation caused by infection, confirmed by chest X-ray or CT scan. Include pneumonias thought to be caused by aspiration of feed or fluid in to the lungs
Pneumonia, hospital acquired	Pulmonary inflammation caused by infection, confirmed by chest X-ray or CT scan. Include all pneumonias other than aspiration pneumonias
Pneumothorax	Presence of gas in the pleural space, confirmed by chest X-ray or CT scan
Pulmonary embolus	Include pulmonary emboli (PE) confirmed by CT pulmonary angiogram (CTPA) or ventilation/perfusion (V/Q) scans
Pulmonary oedema	Fluid accumulation in the lung parenchyma, confirmed by chest X-ray or CT scan
Surgical	
Abscess	Collection of fluid containing pus. Include any intra-abdominal or intrapelvic abscess, detected clinically, by ultrasound or CT scan and/or intraoperatively
Anastomotic leak	Include all anastomotic leaks. Include leaks detected by CT scan and/or intraoperatively; and leaks managed conservatively or surgically
Bile duct injury	Intraoperative injury to the bile ducts requiring further postoperative management
Bile leak	Include all bile leaks. Include leaks detected by CT scan and/or intraoperatively; and leaks managed conservatively or surgically
Bladder injury	Intraoperative injury to the bladder requiring further postoperative management
Chylothorax	Presence of lymphatic fluid in the pleural space, confirmed by chest X-ray or CT scan
Clostridium difficile	*C. difficile* infection must be confirmed by detection of *C. difficile* toxin in faeces
Enterotomy	Accidental surgical incision in to the bowel. Include leaks from enterotomies detected by CT scan and/or intraoperatively; and leaks managed conservatively or surgically
Haematoma	Collection of fluid-containing blood, diagnosed clinically or by ultrasound or CT scan
Haemorrhage, reactionary	Haemorrhage from operative sites within 48 h of operation
Haemorrhage, secondary	Haemorrhage from operative sites after 48 h of operation
Ileus	Delay to return to normal gut function, defined as intolerance to solid food and/or failure to pass flatus >3 days following operation
Ischaemic colitis	Inflammation of the colon caused by inadequate blood supply, diagnosed clinically, by CT scan and/or intraoperatively
Postoperative nausea	Postoperative nausea requiring intervention
Seroma	Collection of serous fluid, diagnosed clinically or by ultrasound or CT scan
Splenic injury	Intraoperative injury to the spleen requiring further postoperative management
Upper gastrointestinal (upper GI) bleed	Include upper GI bleed of any aetiology other than haemorrhage from operative sites (select ‘haemorrhage, reactionary/secondary’ for these)
Ureteric injury	Intraoperative injury to the ureters requiring further postoperative management
Wound dehiscence	Rupture of a surgical wound along the suture line
Wound infection	We advise adherence to the Centre for Disease Control's definition of surgical site infection, which is any one of: Purulent drainage from the incision At least two of: pain or tenderness; localised swelling; redness; heat; fever; AND The incision is opened deliberately to manage infection or the clinician diagnoses a surgical site infection Wound organisms AND pus cells from aspirate/swab
Miscellaneous	
Blood stream infection	An infection not related to infection at another site, with a recognised pathogen cultured from blood cultures which is not related to an infection at another site
Cellulitis	Bacterial infection involving the skin
Central line infection	Infected peripherally inserted central catheter (PICC) or central lines, confirmed by culture of line tip
Fracture	Any fracture sustained postoperatively, diagnosed by plain film X-ray, CT or MRI
Peripheral line infection	Localised cellulitis (erythaema and swelling) around a peripheral cannula insertion site
Pressure sore	Decubitus ulcers, localised injuries to the skin and/or underlying tissue as a result of pressure usually over a bony prominence
Other	*Please enter free text*

### Explanatory variables

BMI is the main explanatory variable. Patients will be stratified by BMI; underweight (BMI <18.5), healthy weight (BMI 18.5–24.99), overweight (BMI 25–29.99) and obese (BMI ≥30). The American Society of Anaesthesiologists (ASA) score will be recorded for each patient to adjust for global comorbidity status. Smoking history will be recorded and the Revised Cardiac Risk Index will be calculated for each patient to adjust for pre-existing cardiovascular risk (box 1).^17^ The Malnutrition Universal Screening Tool (MUST)^18^ and Nutritional Risk Index^19^ will be calculated to adjust for nutritional status. The Index of Multiple Deprivation (IMD) will be recorded to adjust for socioeconomic status. Procedures will be classified according to their operative complexity as per the British United Provident Association (BUPA) schedule of procedures.^17^

Box 1 **The revised cardiac risk index**

Revised Cardiac Risk Index

1. History of ischaemic heart disease
2. History of congestive heart failure
3. History of cerebrovascular disease (stroke or transient ischaemic attack)
4. History of diabetes requiring preoperative insulin use
5. Chronic kidney disease (creatinine >177 mmol/L)
6. Undergoing suprainguinal vascular, intraperitoneal, or intrathoracic surgery

### Patient identification and data collection

Collaborators will be asked to screen operating lists for eligible patients daily or as frequently as is practically possible. The patient demographics and operative data fields should be completed as soon as the patient is identified as being eligible to be included in DISCOVER. Collaborators will be encouraged to regularly monitor patients for complications in the postoperative period. The follow-up fields should be collected as soon as possible after the end of the 30-day follow-up.

### Quality assurance

Although, many collaborators participating in the study will be medical students, each local team must include at least one qualified doctor to oversee and supervise the students. The study will additionally be registered with a sponsoring consultant surgeon at each hospital site.

A detailed protocol describing how to register and run the study will be made available to collaborators online and by email. This will describe in detail the inclusion and exclusion criteria, along with examples. Possible follow-up strategies will be discussed. The principles underlying the Clavien-Dindo classification will be explained with examples. The protocol will also include an indepth description of data fields and the potential data sources which collaborators could use to collect them. The protocol will be interactively presented at a national collaborator meeting and at local collaborator meetings organised by the study's regional leads. Feedback from these meetings will be used to clarify any ambiguities in the protocol.

To overcome the learning curve in identifying patients and relevant data, all participating centres will be asked to pilot completing patient identification and the initial stages of the data collection form for 1 day in the week leading up to the main study's starting date.

To ensure collaborators understand the inclusion criteria and application of the Clavien-Dindo classification, they will be asked to complete a case-based online e-learning module prior to starting data collection.

To ensure that the primary outcome is accurately recorded, the Clavien-Dindo grade for each complication experienced by a patient will be independently assessed by two collaborators. Any disagreements will be resolved by discussion with other members or supervisors.

Throughout the data collection period, the trial management group will hold weekly Twitter question and answer sessions (https://twitter.com/STARSurgUK), giving the opportunity for collaborators to clarify any uncertainties regarding the protocol. A summary of frequently asked questions will be distributed to all collaborators following each Twitter session, providing near real-time feedback to collaborators.

### Validation

Following data collection, only data sets with >95% data completeness will be accepted for pooled national analysis. An independent assessor will validate 5% of all data points, with a target of >95% case ascertainment and >98% data accuracy.

### Data management

Data will be collected and stored online through a secure server running the Research Electronic Data Capture (REDCap)^20^ web application hosted at the University of Edinburgh. REDCap is a secure, web-based application designed to support data capture for research studies by providing: (1) an intuitive interface for validated data entry; (2) audit trails for tracking data manipulation and export procedures; (3) automated export procedures for seamless data downloads to common statistical packages; and (4) procedures for importing data from external sources. It is widely used internationally by academic organisations to store research databases. Collaborators will be given secure login details, including a password for the REDCap project server. All transmission and storage of web-based information by this system is encrypted. Any patient identifiable information will not be available for data-analysis and will be automatically stripped from the database when exported from REDCap.

### Anticipated minimum recruitment

It is estimated that an average centre performs approximately 40 gastrointestinal and hepatobiliary cases, thus meeting the study inclusion criteria in a 28-day period. A minimum of 148 centres will be recruited, with at least four centres participating at each of 37 medical schools. Overall, we anticipate recruiting at least 5920 patients in total.

### Power calculation

This study is powered to detect a significant difference between obese patients (BMI ≥30) and patients with healthy weight (BMI 18.5–24.99). A total of 3550 patients would provide 80% power to detect a 35% increase in the postoperative complication rate from 8% to 10.8% (α=0.05, matched 1 experimental (n=1775): 1 control (n=1775), power=0.80).

### Statistical analysis

Differences between demographic groups will be tested with the χ^2^ test. Multivariable binary logistic regression will be used to test the influence of clinically plausible variables on the outcome measures, to produce adjusted ORs and bootstrapped 95% CIs. This will be performed first on the whole dataset and then a matched group of 1:1 control (healthy weight): experimental (obese), using propensity scoring. Data handling will be performed in SPSS V.21.0 and statistical modelling in the R Foundation Statistical Programme V.3.0.0.

## Ethics and dissemination

### Research ethics approval

Following review of the study protocol by a Research Ethics Committee Chairperson and a University NHS Trust Research & Development Office Director, the authors were advised that this observational study can be undertaken as a clinical audit and does not require formal ethical review. Caldicott guardian approval was granted to store patient data. This study will be registered as clinical audit or service evaluation at each participating hospital.

### Protocol dissemination

The protocol will be disseminated primarily through medical student networks, including student surgical and medical societies. Postgraduate research collaboratives and the Association of Surgeons in Training (http://www.asit.org) will also disseminate the protocol to their members. A student local lead will be designated at each medical school to facilitate local dissemination. The protocol document will be made available online and will also be disseminated through social media, including Twitter (https://twitter.com/STARSurgUK) and Facebook (https://www.facebook.com/STARSurgUK). The novel use of social media to drive collaborator recruitment by the STARSurg collaborative has been described previously.^21^

## Discussion

The study described in this protocol will assess the health needs of an increasing population of surgical patients for whom current surgical outcome data are conflicting. It will provide a prospective snap-shot to inform priorities in the perioperative management of obese surgical patients. Should obese patients be found to be at increased risk of postoperative complications, this would demonstrate a need for the development of novel interventions to reduce this risk.

This study has been designed to be delivered through a national student network, with the aim of engaging students with multicentre studies. To maximise recruitment to the study it has been designed to be registered as clinical audit or service evaluation. To facilitate this, a pragmatic approach was adopted, most importantly ensuring that the study is purely observational. The project's complexity does warrant a detailed protocol to ensure consistency and reproducibility across all the centres participating in data collection.

The limitations of this study relate to the observational methodological design. Being unable to proactively gather anthropometric data, we expect that BMI values will be unavailable for a proportion of patients. Equally it is inevitable that there will be some missing data; however, only 1.5% of patients in our previous collaborative study had missing data.^11^ To attempt to maximise data completeness, regular reminders will be sent to collaborators with ongoing assistance from supervisors to ensure available data is not missed.

Collaborators will rely on clear documentation of complications in the medical notes and discharge letters to identify morbidity in the follow-up period. Since minor complications are not always consistently documented, it is likely that DISCOVER may underestimate the incidence of some complications. However, DISCOVER will offer a comprehensive overview of all postoperative events and this depth of data will overcome some of these limitations.

An observational study is unlikely to definitively prove causation between obesity and morbidity; however, DISCOVER is likely to present the best quality of evidence available on this topic. Importantly, DISCOVER will generate the data necessary to power any future clinical trials aiming to provide grade 1 evidence in this arena.

This project will aid the continued development of the STARSurg collaborative network, with the addition of more centres, including hospitals in the Republic of Ireland. As the network continues to mature, it will develop the infrastructure to deliver interventional studies whose design would be informed by this observational study.
